# Linkage and Demographic Indexing for Nursing Dynamics in the Integrated Data Service

**DOI:** 10.23889/ijpds.v9i5.2828

**Published:** 2024-09-18

**Authors:** Nathan O'Connor, Tolu Adedire, Dilly Stephenson, Leah Quinn

**Affiliations:** 1 Office for National Statistics

## Objective and Approach

This project links UK Nursing and Midwifery Council (NMC) Registrant data to the 2021 England and Wales Census (“Census 21”).

The Office for National Statistics (ONS) has composed a framework of index tables to facilitate linkage between prominent datasets in the UK. In linking person-level data to Census 21, ONS’ Demographic Index enables onward linkage to other administrative datasets that are indexed alike.

NMC Register and Census 21 records have been initially linked using bespoke deterministic and probabilistic methods, with resulting matches being joined to the Demographic Index. ONS’ Demographic Index Matching Service (DIMS) will also be used to link NMC data to the Demographic Index to enable comparisons between methods.

## Results

Analysis of both approaches will compare quality metrics such as match rate, recall and precision and tools will be used to assess bias in matches made through each method. These metrics will provide an insight into how the index matching services have been designed and whether improvements can be made to account for any shortcomings in their performance.

## Conclusions

Hybrid approaches using both bespoke and indexing methods are proving valuable where sole use of the matching services is not suitable. This research will help ONS to refine which methods will yield the highest quality linkage and when.

## Implications

As ONS pursues an ‘indexing first’ approach to linkage, the outcomes of this and similar projects will influence the future of utilising indexes in varied capacities when creating or maximising the potential of linked data.

